# Functional Orthodontic Rehabilitation and Craniofacial Growth in Pediatric Mandibular Condylar Fractures: A Narrative Review

**DOI:** 10.3390/children13101425

**Published:** 2026-10-17

**Authors:** Lucia Giannini, Francesca Gaffuri, Giovanni Cattaneo, Cinzia Maspero

**Affiliations:** 1 Fondazione IRCCS Ca’ Granda Ospedale Maggiore Policlinico, 20122 Milan, Italy; cinzia.maspero@unimi.it; 2 Department of Biomedical, Surgical and Dental Sciences, University of Milan, 20122 Milan, Italy

**Keywords:** mandibular fractures, mandibular condyle/injuries, child, adolescent, bone remodeling, orthodontic appliances, functional, fracture fixation, internal, temporomandibular joint

## Abstract

**Highlights:**

**What are the main findings?**

- Observational evidence suggests substantial condylar remodeling and functional recovery in many growing patients when comfortable mandibular mobility is restored and prolonged immobilization is avoided.
- Management ranges from expectant and functional approaches to selective ORIF; no single strategy is universally superior across pediatric fracture patterns.

**What are the implications of the main findings?**

- Treatment should be individualized according to skeletal maturity, fracture displacement, occlusion, mandibular mobility, cooperation, and remaining growth.
- Long-term orthodontic and craniofacial surveillance is essential to detect evolving asymmetry, malocclusion, TMJ dysfunction, or ankylosis during growth.

**Abstract:**

Background/Objectives: Pediatric mandibular condylar fractures require management that accounts for ongoing craniofacial growth and temporomandibular joint (TMJ) function. This narrative review summarizes contemporary evidence on age- and displacement-sensitive decision-making, functional and orthodontic rehabilitation, imaging, and long-term growth surveillance. Methods: A structured narrative search of PubMed/MEDLINE, Scopus, Web of Science, and the Cochrane Library was conducted for English-language human studies published from January 2021 through June 2026, supplemented by citation searching for foundational publications. Results: Management ranges from expectant observation and functional appliance therapy to brief closed stabilization and open reduction with internal fixation (ORIF). Predominantly retrospective and heterogeneous evidence suggests that timely restoration of comfortable mandibular mobility is associated with favorable functional recovery and substantial remodeling potential in many growing patients, although outcomes depend on age, fracture morphology, functional loading, and remaining growth. ORIF may be appropriate for selected severe fractures when acceptable function and occlusion cannot be restored by less invasive measures. Conclusions: Management should integrate skeletal maturity, fracture level and displacement, laterality, occlusal disturbance, mandibular mobility, cooperation, and remaining growth. Across treatment modalities, avoidance of unnecessary prolonged immobilization, structured functional rehabilitation, and longitudinal orthodontic and craniofacial surveillance are important clinical priorities.

## 1. Introduction

Fractures of the mandibular condyle are disproportionately common in children, reflecting the anatomy and biomechanics of the developing craniofacial skeleton. Across pediatric maxillofacial trauma series, the condylar region is consistently identified as a frequent mandibular fracture site, with reported proportions varying according to population and diagnostic criteria [1,2,3,4]. The relatively large condylar head, thin cortical bone, and narrow, highly vascularized neck contribute to vulnerability after indirect trauma to the chin or mandibular body [1].

Unlike body or symphyseal fractures, which generally follow direct impact, condylar injuries commonly arise when kinetic energy transmitted along the mandibular arch concentrates at the condylar neck or head. In children, this indirect loading commonly follows a fall onto the chin or symphysis, a lateral blow to the mandibular body or angle, or higher-energy bicycle, sports, or motor-vehicle trauma [1,2,3]. Because the condyle is an important site of adaptive endochondral growth, the injury is not only a problem of reduction and stabilization but also a potential developmental disturbance. In younger patients, rich vascularity and active periosteal and cartilaginous tissues support rapid healing and substantial adaptive remodeling, whereas intra-articular injury, prolonged immobility, or an unfavorable functional environment may promote restriction or ankylosis; this biological balance supports growth-aware selection among expectant, functional, closed, and surgical treatment [1]. Delayed diagnosis or inappropriate immobilization may contribute to progressive facial asymmetry, malocclusion, limited mandibular function, or TMJ ankylosis, particularly in preverbal or uncooperative children.

Despite extensive clinical experience, an international consensus on pediatric condylar fracture management remains elusive. A multicenter study involving 14 maxillofacial units demonstrated marked variability from expectant care to maxillomandibular fixation (MMF) and ORIF, with institutional practice and surgeon preference influencing treatment selection [2]. Systematic reviews likewise identify heterogeneous classification systems, indications, rehabilitation protocols, and outcome definitions, with limited high-certainty comparative evidence [5,6].

The 2024 narrative review by Bottini et al. provided a broad overview of the biological principles and principal management options for pediatric mandibular condylar fractures [1]. The present review complements that work by placing greater emphasis on functional orthodontic rehabilitation, radiation-conscious diagnostic pathways, longitudinal craniofacial growth surveillance, and the integration of these domains into a clinically oriented decision framework.

## 2. Materials and Methods

A structured narrative literature search was conducted in PubMed/MEDLINE, Scopus, Web of Science, and the Cochrane Library for English-language human studies on pediatric mandibular condylar fractures published from January 2021 through June 2026. Search concepts combined terms related to the mandibular condyle, fracture, child, pediatric age, adolescence, diagnosis, treatment, rehabilitation, orthodontics, and outcomes. Animal studies, in vitro investigations, finite element analyses, adult-only cohorts, and reports without clinically relevant diagnostic, therapeutic, rehabilitation, or outcome information were not included in the core evidence synthesis. Database searching was supplemented by backward citation searching of relevant reviews and eligible articles to identify foundational publications on classification, craniofacial growth, and long-term outcomes. No separate systematic search of the grey literature was undertaken.

Historical sources outside the contemporary search window were retained only when they met at least one explicit contextual criterion: they were the original description of a classification or terminology still used in current practice; they reported pediatric growth or functional outcomes across prolonged follow-up; or they described a treatment concept not adequately represented in the contemporary literature. These publications were used to provide historical or developmental context and not to estimate contemporary comparative effectiveness.

Because this article was designed as a narrative rather than a systematic review, study selection was guided by clinical relevance to the predefined domains of epidemiology, diagnosis, classification, management, rehabilitation, complications, and long-term growth. Titles and abstracts identified through the database searches were reviewed for relevance, and potentially informative full texts were examined for inclusion in the narrative synthesis. Greater weight was given to multicenter studies, systematic reviews, longitudinal cohorts, comparative clinical series, and long-term follow-up reports; case reports were retained when they illustrated an uncommon technique or an unusually long developmental trajectory. No formal dual-reviewer screening procedure, risk-of-bias assessment, or quantitative pooling was planned. The structure and reporting were informed by the Scale for the Assessment of Narrative Review Articles (SANRA) [7].

For operational purposes, a report was considered clinically relevant when it included children or adolescents (or an extractable pediatric subgroup), addressed at least one predefined review domain, and reported information capable of informing diagnosis, treatment selection, rehabilitation, or surveillance, such as fracture pattern, occlusion, mandibular mobility, pain or TMJ symptoms, complications, craniofacial growth, or need for further intervention. Prioritization reflected breadth and directness of evidence—multicenter sampling, clearly defined pediatric cohorts, comparative design, and longitudinal follow-up—rather than an assumption that a design label alone ensured quality. Smaller single-center series and case reports were not excluded solely because of lower evidentiary level; they were used selectively for uncommon techniques or unusually long developmental trajectories, and their findings were not generalized beyond their design. To broaden source capture beyond higher-level comparative designs, eligibility was not restricted to multicenter studies: single-center cohorts, smaller clinical series, case reports, and studies with an extractable pediatric subgroup were also considered when they directly informed a predefined review domain.

The evidence is therefore presented as a structured narrative synthesis. Table 1 provides a representative clinical evidence map rather than an exhaustive list of all publications considered. A final verification search was performed in September 2026 using the same concepts to identify eligible reports published within the predefined January 2021–June 2026 period; the recent studies by Scheibl et al. (2024) [8] and Rador et al. (2026) [9] were incorporated into the evidence map and narrative synthesis.

## 3. Characteristics of the Evidence Base

The evidence base considered in this review was characterized by marked heterogeneity in study design, age distribution, fracture classification, treatment protocols, follow-up duration, and outcome definitions, and was dominated by retrospective cohorts and heterogeneous reviews with few prospective comparative studies.

Study design alone does not establish methodological quality, and apparent differences between expectant, functional, closed, and surgical strategies remain susceptible to confounding by indication.

## 4. Epidemiology and Etiology

Although absolute injury rates vary by geography, socioeconomic conditions, and case ascertainment, the relative contribution of condylar injury to pediatric mandibular trauma is substantial. Contemporary series report bilateral involvement in approximately one-fifth of pediatric condylar fracture cases, while boys are generally over-represented, with reported male-to-female ratios of approximately 1.8:1 to 2.6:1 [1,2,3,4]. Because these percentages use different denominators, Table 2 explicitly states the relevant reference population.

The dominant mechanism changes with age:

- Infants and preschool children (<6 years): low-velocity falls from standing height, furniture, or playground equipment predominate. Facial fractures are comparatively uncommon because of the high cranium-to-face ratio, greater osseous elasticity, and generous soft-tissue envelope; when mandibular trauma occurs, the condyle is particularly vulnerable.
- School-age children and adolescents: bicycle accidents, sports trauma, and motor-vehicle collisions become more prominent.
- Older adolescents: interpersonal violence contributes an increasing proportion of injuries, particularly among males.

### Non-Accidental Injury: A Diagnostic Alert

In a young child with facial trauma and an unclear, inconsistent, or evolving history, clinicians should maintain a low threshold for considering non-accidental injury (NAI). In a National Trauma Data Bank cohort of 9741 children aged 5 years or younger with facial fractures, 193 cases (2.0%) were classified as non-accidental trauma. Within this facial-fracture cohort, mandibular fractures were more frequent in NAI than accidental cases (38.9% vs. 21.1%); among patients with mandibular fractures, condylar involvement was also more frequent (75.8% vs. 48.4%) [23]. This pattern should increase clinical suspicion only in the appropriate context and does not, by itself, establish abuse. A focused study of infants and toddlers similarly found that an isolated mandibular fracture after a clearly described short fall may be accidental, whereas inconsistent history, occult injuries, or non-craniofacial cutaneous findings should prompt formal safeguarding assessment [24]. Oral health and emergency clinicians are well positioned to recognize orofacial sentinel injuries, including bruising, intraoral trauma, and unexplained fractures, and to initiate the appropriate referral pathway [25].

## 5. Condylar Biology and Craniofacial Growth

The mandibular condyle is not a static articular surface. Its secondary cartilage contributes to adaptive endochondral growth, ramus height, and sagittal and vertical facial development. In children, the condylar head is proportionally larger and more vascular, is covered by highly osteogenic tissues, and sits above a relatively narrow neck.

This anatomy creates two clinically important and apparently opposing properties (Table 3):

### The Remodeling Double-Edged Sword

Substantial remodeling capacity underlies the preference for nonsurgical management in many young children. Under appropriate functional conditions, displaced fractures may undergo considerable adaptive remodeling, but the extent and clinical adequacy of remodeling are variable and depend on age, fracture level and displacement, disc-capsule integrity, functional loading, and remaining growth [1,9,11,14]. A meta-analysis of functional appliance treatment reported favorable dimensional recovery in 223 growing patients [11], while a retrospective series of 108 children with articular-impact fractures documented satisfactory functional recovery and a low frequency of ankylosis [14]. A 2026 retrospective series of 60 patients (75 fractures) managed with closed treatment likewise reported restitutional remodeling in all fractures, although minor complications occurred in 20% of patients [9]. A separate systematic review reported generally favorable functional and imaging outcomes after functional orthodontic therapy, while noting that residual deviation may persist after unilateral or luxated injuries [12].

Long-term case observations illustrate, but do not prove, developmental trajectories. Farronato et al. reported 12 years of stable occlusion, pain-free TMJ function, facial symmetry, and radiographic remodeling after conservative functional appliance therapy and physiotherapy [21]. A 20-year follow-up from the same clinical school documented sustained mandibular function and facial symmetry after appropriately indicated open treatment and subsequent rehabilitation [22]. These reports cannot establish comparative effectiveness, but they show that favorable growth may follow different treatment pathways when function is restored and surveillance is maintained.

Remodeling is not synonymous with guaranteed anatomical restitution. Fracture level, displacement, integrity of the disc-capsule complex, functional loading, age, and remaining growth influence the result, and clinical recovery may not parallel radiographic symmetry. The same osteogenic and articular reactivity that supports remodeling can promote fibrous or bony ankylosis after comminution, delayed treatment, or prolonged immobilization [1]. Mandibular restriction is not synonymous with ankylosis: it may also arise from pain, muscle guarding, edema, disc displacement, mechanical obstruction, or periarticular scarring. Post-traumatic fibrosis refers to scar formation within or around the capsular tissues and may restrict movement without true joint fusion. Fibrous ankylosis, in contrast, refers to dense restrictive intra-articular adhesions, whereas bony ankylosis involves osseous bridging or fusion; their diagnosis, severity, and management therefore differ [1].

Age-related outcome data require caution. In a historical cohort of pediatric mandibular fractures—not an isolated condylar-fracture cohort—orthognathic correction was reported in 22% of children injured at 4–7 years and 17% of those injured at 8–11 years, while it was uncommon after injuries before age 4 or after age 12 [20]. These data support prolonged growth surveillance but should not be interpreted as condylar-fracture-specific risk estimates or a universal biological threshold.

## 6. Classification Systems

Clinically useful classification should document anatomical level using consistent terminology (condylar head/diacapitular region, neck, and base or subcondylar region), fragment relationship, condyle-fossa position, laterality, associated mandibular injuries, occlusion, and mandibular mobility, interpreted in the context of dentition and skeletal maturity. The Lindahl, Loukota, and AO/CMF frameworks remain widely used [26,27,28]; however, their pediatric-specific validation is limited, and cross-study application is not uniform.

### 6.1. Classic and Refined Frameworks

Lindahl classification. This system stratifies fractures according to anatomical level (condylar head, neck, or subcondylar/base region), fragment relationship and displacement, and the position of the condylar head relative to the fossa. Its original cohort also demonstrated age-related differences in angulation and segment overlap [26].

Loukota classification. This framework refined the anatomical terminology for high condylar injuries by distinguishing diacapitular fractures involving the condylar head from fractures of the neck and base/subcondylar region. The distinction is clinically relevant because intra-articular injury may increase the risk of joint stiffness or ankylosis [27].

AO/CMF comprehensive classification. The level-3 system further describes anatomical level, displacement, dislocation, and fracture morphology, providing a standardized language for clinical reporting and research [28].

### 6.2. Pediatric Treatment-Relevant Variables

A pediatric position-displacement classification stratifies injuries into condylar head fractures (type A), mildly displaced neck/base fractures (type B), and severely displaced neck/base fractures (type C), relating fracture morphology to proposed treatment pathways [10]. Pediatric interpretation should additionally consider chronological and skeletal age, dentition, laterality, involvement of the articular or growth-related region, degree and direction of overlap, baseline occlusion, developmental cooperation, and the ability to restore stable function. No classification system has been universally adopted or extensively validated specifically for pediatric outcome prediction, which continues to limit cross-study comparison and precise evidence-based treatment thresholds [5,6,10].

The absence of a universally accepted pediatric classification also impedes the validation and reproducibility of treatment algorithms and limits the generalizability of findings across centers and populations.

## 7. Clinical and Radiological Diagnosis

A condylar fracture should be considered after trauma to the chin, mandibular body, or angle, because energy may be transmitted indirectly along the mandibular arch to the condylar neck or head. In preverbal or uncooperative children, the primary impact site can distract attention from the joint injury.

### 7.1. Clinical Examination

Typical findings include:

- Preauricular pain, edema, or focal tenderness;
- Painful or restricted opening, reduced or asymmetric condylar translation, and deviation toward the injured side;
- Ipsilateral premature contact and contralateral open bite in unilateral injuries; anterior open bite after bilateral injury;
- Refusal to eat, irritability, drooling, guarding, or a chin wound in a young child who cannot localize TMJ pain.

These patterns are suggestive rather than diagnostic and should be interpreted against pre-injury occlusion and associated dentoalveolar trauma. Baseline documentation should include maximal comfortable opening, excursions, facial symmetry, midline position, and occlusion whenever cooperation permits.

A targeted history should document the mechanism and circumstances of injury, time elapsed since trauma, current symptoms, relevant medical and dental history, preinjury occlusion, and baseline mandibular function. In pediatric patients, the reported mechanism should be assessed for consistency with the clinical and radiographic findings, while recognizing that no single fracture pattern is diagnostic of abuse [23,24].

### 7.2. Imaging Selection

Imaging should answer a defined clinical question. Panoramic radiography can contribute to initial screening when cooperation is adequate and two-dimensional information is sufficient, but projection overlap limits characterization of intracapsular injury and precise displacement. CT or limited-field cone-beam CT (CBCT) provides superior three-dimensional osseous definition of fracture level, displacement, dislocation, ramus height, associated injuries, and mechanical obstruction when this information will alter management; CT has broader trauma and soft-tissue capability, whereas CBCT is primarily an osseous examination with protocol-dependent radiation dose. MRI is not a substitute for acute osseous fracture characterization but is complementary when disc position, cartilage, joint effusion, capsular injury, or persistent unexplained symptoms are clinically important. Pediatric field of view and exposure settings should be optimized, and repeat ionizing imaging should not be routine [13,29] (Table 4).

MRI is complementary when disc position, cartilage, joint effusion, capsular injury, or persistent unexplained symptoms are clinically important. A systematic review supports its selective use in maxillofacial trauma [29], and MRI follow-up after functional treatment demonstrates that favorable mandibular function and remodeling may coexist with residual disc abnormalities [13].

## 8. Management: A Growth-Oriented Decision Framework

Four broad strategies are used: expectant care, functional/orthodontic rehabilitation, brief closed stabilization, and ORIF. No approach has demonstrated categorical superiority in prospective randomized pediatric trials. Treatment should therefore integrate chronological and skeletal age, dentition, fracture level and displacement, laterality, occlusal stability, mandibular mobility, associated injuries, remaining growth, patient cooperation, and local expertise [2,5,6]. Recent comparative observational data remain hypothesis-generating rather than definitive: in a 28-patient retrospective pilot study, closed treatment was associated with better six-month mandibular range-of-motion outcomes after multivariable adjustment, but the small sample, age imbalance, treatment-selection bias, and wide confidence intervals limit causal interpretation [8] (Table 5).

**Figure 1:**
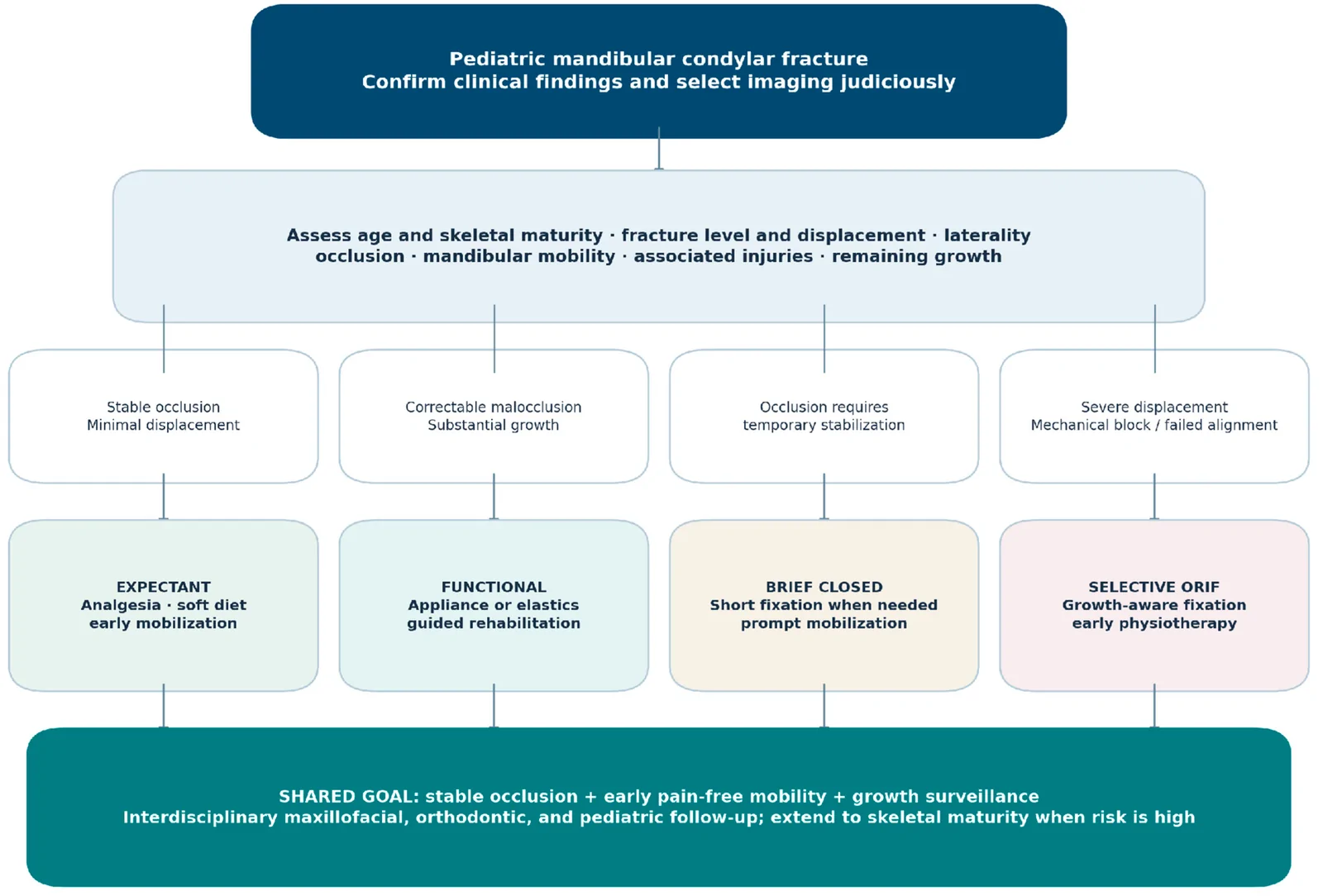
Proposed clinical decision pathway for pediatric mandibular condylar fractures. The pathway summarizes narrative evidence and expert clinical reasoning; it has not been prospectively validated. ORIF, open reduction and internal fixation.

Figure 1 summarizes the proposed clinical decision pathway from diagnosis through treatment selection and growth surveillance.

### 8.1. Expectant Management

Expectant care is appropriate when the fracture is nondisplaced or minimally displaced, occlusion is stable or readily reproducible, and mandibular movement can resume without a mechanical block. Care includes analgesia, temporary dietary modification, reassurance, early tolerated mobilization, and scheduled review. The absence of an appliance should not be mistaken for absence of treatment: monitoring and timely restoration of comfortable movement are central [1,14]. Its principal advantages are avoidance of appliance- and procedure-related morbidity and preservation of joint motion; its limitations are dependence on stable or readily reproducible occlusion, reliable caregivers, and close reassessment, because loss of occlusion or persistent restriction may require escalation.

### 8.2. Functional and Orthodontic Rehabilitation

Guiding elastics, acrylic splints, activators or other functional appliances, and structured mandibular exercises can re-establish intercuspation while permitting joint motion and functional loading. This strategy is particularly relevant in growing mixed-dentition patients with mild-to-moderate occlusal derangement and preserved potential for guidance. Evidence from systematic reviews supports favorable clinical and dimensional recovery, while emphasizing low certainty and heterogeneous protocols [11,12]. Functional care combines occlusal guidance with continued joint loading and avoids open surgery; however, its effectiveness depends on cooperation, appliance fit and adherence, oral hygiene, and the absence of an irreducible mechanical block, and published protocols remain heterogeneous.

CAD/CAM thermoformed splints can individualize occlusal guidance during closed or functional care [15]. Fiber-reinforced composite splints with ligature wire have been described as a low-cost option for pediatric mandibular stabilization [16], and a recent case report documented recovery of function and occlusion after use of a function-generating bite appliance [17]. These techniques should be selected according to dentition, cooperation, fracture stability, and clinician expertise rather than treated as interchangeable devices.

### 8.3. Closed Treatment and Maxillomandibular Fixation

MMF may be useful when occlusion cannot initially be maintained without temporary support and the dentition permits safe anchorage. Techniques include selected arch bars, wires, screws, or guiding elastics, with attention to developing teeth and soft tissues. In growing patients, rigid immobilization should be as short as clinically feasible and followed promptly by elastic guidance and active mobilization. Published pediatric protocols commonly describe brief periods, often about 7–14 days, but no universally validated duration exists; the decision must reflect stability, symptoms, and joint-stiffness risk [1,2]. Closed stabilization offers rapid temporary control of occlusion without surgical exposure, but rigid or prolonged MMF can compromise nutrition and oral hygiene, risk injury to developing teeth or periodontal tissues, and increase joint stiffness; airway safety and the ability to release fixation are additional practical concerns [1,2,6].

### 8.4. Open Reduction and Internal Fixation

ORIF is not the default for a young child whose occlusion and function can be restored less invasively, but chronological age is not an absolute contraindication. Consideration is appropriate for severe displacement or dislocation with persistent functional or occlusal impairment, mechanical obstruction, open or comminuted injury, associated unstable fractures, or failure of a carefully supervised nonsurgical approach [2,5,6]. In adolescents nearing skeletal maturity, indications progressively resemble adult practice. Its principal advantage is direct restoration of fracture anatomy, ramus height, and occlusion when these cannot be recovered functionally; disadvantages include anesthetic and surgical morbidity, facial nerve and scar risk, hardware-related complications or removal, and uncertainty regarding growth-related effects [5,18,19,30].

Surgical planning should account for thin pediatric bone, developing teeth, the facial nerve, the condylar growth-related region, and possible hardware removal. A pediatric series of 14 fractures in 12 children aged 3–11 years demonstrated that transoral endoscopic-assisted reduction and hardware removal are feasible in experienced hands [18]. A separate endoscopic case report and review supports the potential to reduce external scarring and facial nerve exposure in selected patients, while underscoring the learning curve and need for specialized equipment [30].

Potential benefits of ORIF include restoration of ramus height and occlusion when a displaced fragment cannot be managed functionally. Risks include facial nerve injury, scarring, infection, hardware problems, and uncertain effects on remodeling. A 2025 retrospective cohort published in Children found no statistically significant difference in complication rates between conservative and surgical groups, but only six patients underwent surgery [19]. The absence of a statistically significant difference in such an imbalanced retrospective comparison should not be interpreted as evidence of equivalence between treatment strategies, particularly because confounding by indication is likely. Similarly, the 28-patient retrospective pilot study by Scheibl et al. found associations favoring closed treatment for several six-month functional outcomes, but its limited sample and non-randomized treatment selection preclude definitive comparative conclusions [8]. Surgical technique and rehabilitation should therefore receive the same attention as the decision to operate.

## 9. Complications and Long-Term Sequelae

Because injury occurs in an actively developing skeleton, sequelae may emerge gradually after apparently satisfactory early recovery. Favorable outcome depends on timely diagnosis, restoration of mandibular mobility, stable occlusion, and follow-up sufficient to detect developmental change (Table 6).

### 9.1. Growth Disturbance, Malocclusion, and Facial Asymmetry

Potential developmental findings include shortened ramus height, mandibular midline deviation, crossbite, occlusal-plane cant, facial asymmetry, retrognathia, and restricted movement. Residual radiographic irregularity can coexist with clinically acceptable occlusion and function [9,11,12,14,21]. Functional outcomes should also be documented explicitly, including pain, maximal opening, lateral and protrusive excursions, deviation, chewing function, and patient-reported TMJ symptoms; contemporary comparative data confirm that range-of-motion outcomes may remain clinically relevant even when major structural complications are absent [8]. Orthodontic assessment should distinguish transient protective posturing from a persistent functional shift or true asymmetric growth. Longitudinal records may include standardized facial photographs, age-appropriate occlusal documentation, maximal opening and excursions, dental eruption, and symmetry. Three-dimensional imaging should be reserved for a defined management question such as progressive asymmetry, suspected ankylosis, or planning for orthodontic-surgical correction.

### 9.2. Temporomandibular Joint Ankylosis

TMJ ankylosis is the most disabling long-term complication. Fibrous or bony fusion can cause severely restricted opening, impaired speech and oral hygiene, micrognathia, and obstructive sleep apnea in bilateral disease [31,32]. Risk is increased by intra-articular injury, hemarthrosis, delayed diagnosis, and prolonged immobilization [1,27,32].

Established ankylosis may require gap or interpositional arthroplasty, reconstruction with a costochondral graft, or selected alloplastic reconstruction, followed by intensive physiotherapy. A systematic review of costochondral graft reconstruction in patients younger than 18 years reported reankylosis in approximately 6%, graft overgrowth in 14%, insufficient growth in 22%, and facial asymmetry in 20%, illustrating unpredictable graft behavior [31]. Radiographic assessment after ankylosis surgery without growth-center transplantation demonstrates variable ramal growth and reinforce the need for long-term monitoring [33]. Prevention strategies therefore emphasize timely restoration of mandibular function and avoidance of unnecessary prolonged immobilization, although the supporting pediatric evidence is predominantly observational [1,14,31,32,33].

### 9.3. Long-Term Follow-Up and Orthodontic Surveillance

Clinical success should not be judged only by union or early pain relief. Follow-up should assess opening and excursions, pain and joint symptoms, reproducible occlusion, mandibular midline, facial symmetry, dental eruption, ramus height or condylar morphology when imaging is justified, and the possible need for orthodontic, reconstructive, or orthognathic intervention. Children with bilateral, intracapsular, markedly displaced injuries or residual asymmetry warrant longer surveillance toward skeletal maturity [14,20,21,22]. If a residual dentofacial deformity ultimately requires combined orthodontic-surgical correction after growth, presurgical orthodontic planning may include occlusal splint guides [34]. The broader orthodontic literature has also examined the relationship between skeletal Class II patterns and temporomandibular disorders and emphasizes the multifactorial nature of TMD rather than a primary causal role of occlusion [35]. This evidence is relevant as contextual background for comprehensive orthodontic and TMJ assessment but should not be interpreted as evidence of fracture-specific causation. Long-term studies that extend follow-up into adulthood after a childhood condylar fracture can provide complementary information on final craniofacial growth, occlusal stability, remodeling, TMJ function, and late treatment requirements; such evidence is informative for developmental trajectories even when it falls outside the primary contemporary search window [21,22].

Long-term surveillance should be multidisciplinary, coordinating maxillofacial, orthodontic and pediatric dental assessment and, when indicated, physiotherapy or pediatric input. Follow-up should integrate occlusal development and facial growth with TMJ symptoms and mobility, condylar remodeling when imaging is clinically justified, and the child’s functional and psychosocial needs (Table 7).

## 10. Controversies and Future Research

Key unresolved questions include:

1. Comparative effectiveness: pediatric treatment guidance relies predominantly on retrospective cohorts and heterogeneous reviews; randomized comparisons are scarce and indication bias is substantial [2,5,6,19].
2. Immobilization parameters: the optimal balance between temporary stabilization for occlusal alignment and early mobilization for ankylosis prevention has not been established prospectively.
3. Surgical indications and technique: long-term, age-stratified data on remodeling, hardware complications, removal, and secondary intervention are needed, including evaluation of minimally invasive access.
4. Outcome standardization: future studies should use consistent pediatric fracture classifications and core outcomes covering occlusion, opening, excursions, symmetry, ramal/condylar morphology, treatment morbidity, secondary intervention, and patient-reported quality of life.
5. Study design: prospective multicenter registries with age-, dentition-, and fracture-stratified reporting may be more feasible than randomized trials for many clinically important questions.

## 11. Strengths and Limitations

This review integrates maxillofacial, functional, orthodontic, and growth-related perspectives rather than focusing only on reduction or surgery. It combines recent systematic reviews and multicenter studies with selected foundational classifications and long-term developmental observations and translates heterogeneous evidence into a practical framework while explicitly avoiding prescriptive thresholds that have not been prospectively validated.

Several limitations should be recognized. This is a narrative rather than systematic review; study selection was guided by clinical relevance, and no formal risk-of-bias assessment or quantitative synthesis was performed. The search was restricted to English-language publications. The available evidence is heterogeneous in age, fracture classification, treatment protocol, follow-up, and outcome definition and is dominated by retrospective observational studies. Some of the longest follow-up evidence derives from single-patient reports. The proposed framework should therefore be understood as a clinically oriented synthesis, not a validated guideline. Although the review domains and source-selection criteria were made explicit, the absence of formal dual-reviewer screening means that subjective selection bias cannot be excluded.

## 12. Conclusions

Pediatric mandibular condylar fractures require a management philosophy that differs from adult trauma because treatment interacts with a growing joint and facial skeleton. Diagnosis should combine careful clinical examination with imaging selected for a defined question and the lowest appropriate radiation burden. Treatment should integrate skeletal maturity, dentition, fracture morphology, laterality, occlusal disturbance, mandibular mobility, cooperation, and remaining growth. Expectant, functional, closed, and surgical approaches may each be appropriate in selected patients; key clinical priorities include restoration of stable occlusion, timely recovery of comfortable mandibular mobility, avoidance of unnecessary prolonged immobilization, and surveillance for ankylosis and growth disturbance. Long-term orthodontic and craniofacial surveillance is particularly important after bilateral, intracapsular, severely displaced, or functionally unstable injuries. A targeted clinical history should include the mechanism and circumstances of trauma, time since injury, symptoms, relevant medical and dental history, preinjury occlusion, and baseline mandibular function, with explicit assessment of concordance between the reported mechanism and the clinical and radiographic findings. Long-term follow-up should be multidisciplinary and should evaluate orthodontic and occlusal development, TMJ function, condylar remodeling, facial symmetry, and craniofacial growth.

## Figures and Tables

**Table 1:** Representative clinical studies informing contemporary management of pediatric mandibular condylar fractures. Sample size is reported as the number of patients unless fractures are explicitly stated.

Study	Design/Sample	Clinical Focus	Key Findings
Bottini et al. (2024) [2]	Multicenter retrospective study; *n* = 180 patients	Management strategies	Marked variability among centers; acceptable outcomes across appropriately selected modalities
Segura-Palleres et al. (2022) [3]	Prospective multicenter study	Epidemiology	The mandibular condyle was the most affected site in children aged <13 years
Chen et al. (2024) [4]	12-year retrospective study	Epidemiology and etiology	Described age-related differences in fracture patterns, etiology, and treatment
Esposito et al. (2024) [5]	Systematic review	Surgical management	ORIF may yield favorable outcomes in selected severe fractures
Jenkyn et al. (2023) [6]	Systematic review	General management	No high-certainty evidence for one universally superior modality
Li et al. (2021) [10]	Pediatric classification study	Classification	Position-displacement classification linked to treatment concepts
Stähli et al. (2021) [11]	Systematic review/meta-analysis; *n* = 223 patients	Functional appliances	Favorable condylar dimensional recovery after functional treatment
Hennig et al. (2024) [12]	Systematic review; 19 studies	Functional orthodontics	Generally favorable functional and imaging outcomes; residual deviation may persist
Sabbagh et al. (2022) [13]	MRI follow-up study	Functional outcomes	Remodeling and ramus-height recovery may coexist with residual disc abnormalities
Malinge et al. (2022) [14]	Retrospective study; *n* = 108 patients	Functional treatment	Good functional recovery and low incidence of ankylosis
Grippaudo et al. (2023) [15]	Clinical study	CAD/CAM splints	Individualized occlusal guidance during closed/functional treatment
Li et al. (2023) [16]	Retrospective clinical study	Conservative splinting	Feasible low-cost stabilization during growth
Leiva Ramirez et al. (2026) [17]	Case report	Functional appliance	Recovery of function and occlusion with a function-generating bite appliance
Abdelazeem et al. (2023) [18]	Retrospective series; *n* = 12 patients/14 fractures	Endoscopic ORIF	Transoral endoscopic reduction and hardware removal were feasible in experienced hands
Ohayon et al. (2025) [19]	Retrospective study; *n* = 71 patients	Conservative vs. surgical care	No significant difference in complication rates; strong group-size imbalance limits comparison
Demianczuk et al. (1999) [20]	Retrospective cohort; *n* = 88 patients	Growth outcomes	Growth-related intervention was concentrated among children injured at 4–11 years
Farronato et al. (2009) [21]	12-year case follow-up	Conservative care	Stable function, occlusion, symmetry, and remodeling across growth
Farronato et al. (2012) [22]	20-year case follow-up	Open treatment	Long-term function and symmetry after appropriately indicated surgery and rehabilitation
Scheibl et al. (2024) [8]	Retrospective pilot study; *n* = 28 patients	Open vs. closed treatment	Closed treatment was associated with better six-month range-of-motion outcomes after adjustment; small sample and non-randomized treatment selection limit inference
Rador et al. (2026) [9]	Retrospective series; *n* = 60 patients/75 fractures	Closed treatment and remodeling	Restitutional remodeling was reported in all fractures; minor complications occurred in 20% of patients

**Table 2:** Approximate distribution of pediatric mandibular fractures reported across contemporary series.

Category	Approximate Distribution	Denominator/Context
Condylar process	40–70%	All pediatric mandibular fractures in reported series
Unilateral condylar fractures	Approximately 80%	Pediatric condylar fracture cases
Bilateral condylar fractures	Approximately 20%	Pediatric condylar fracture cases
Male sex	Male:female ratio approximately 1.8:1–2.6:1	Pediatric condylar fracture patients in reported series
Other mandibular subsites	30–60%	All pediatric mandibular fractures in reported series

**Table 3:** Pediatric condylar anatomy and its clinical implications.

Property	Anatomic Basis	Clinical Implication
Vulnerability to fracture	Narrow neck and thin cortex relative to the condylar head	Acts as a mechanical weak point under indirect impact
High remodeling potential	Vascularity, periosteal response, and active growth	Supports structural adaptation when function and mobility are restored

**Table 4:** Imaging modalities and their roles in pediatric condylar fracture assessment.

Modality	Advantages	Limitations	Clinical Role
Panoramic radiography	Accessible overview; comparatively low radiation burden	Projection overlap; limited intracapsular and soft-tissue detail	Initial screening when cooperation is adequate and two-dimensional information is sufficient; not reliable for precise intracapsular displacement
CT/3D CT	Detailed fracture morphology, displacement, associated trauma, and joint relationships	Ionizing radiation; motion or sedation concerns	Complex injury, major displacement, associated facial trauma, or surgical planning when broader trauma assessment is required
Limited-field CBCT	High-resolution targeted osseous assessment with protocol-dependent dose reduction	Limited soft-tissue information; motion artifacts; dose varies by protocol	Targeted high-resolution osseous assessment when fracture morphology will alter management and broader soft-tissue trauma assessment is unnecessary
MRI	Nonionizing visualization of disc, cartilage, joint fluid, and periarticular soft tissues	Availability, acquisition time, movement, and possible sedation	Complementary soft-tissue assessment for disc/cartilage/capsule questions or persistent symptoms; not first-line for acute bony fracture definition

**Table 5:** Practical framework for age-, occlusion-, and fracture-sensitive treatment selection.

Clinical Presentation	Management Orientation	Key Considerations
Stable occlusion; nondisplaced or minimally displaced injury	Expectant management	Analgesia and soft diet as indicated; early tolerated movement; short-interval reassessment
Correctable malocclusion; preserved movement; meaningful growth remaining	Functional orthodontic guidance	Guiding elastics, functional appliance, or individualized splint; supervised exercises; monitor dentition and symmetry
Occlusion cannot initially be maintained without support	Brief closed stabilization	Use short-duration MMF only when necessary; transition promptly to elastic guidance and active rehabilitation
Severe displacement/dislocation; mechanical block; unstable associated injury; failed nonsurgical alignment	Selective ORIF	Growth-aware fixation and experienced access; protect facial nerve and developing structures; begin rehabilitation early
Intracapsular or bilateral injury; persistent asymmetry; changing occlusion or mobility	Enhanced surveillance	Coordinate maxillofacial and orthodontic follow-up; reassess growth and function; image only when justified

In the largest available multicenter cohort, expectant care was selected in 28% of patients, stand-alone MMF in 26%, and ORIF in 46%. Management differed significantly by fracture morphology and among participating departments, highlighting the continuing influence of institutional practice [2]. Complication rates were low overall, but observational data cannot remove confounding by indication.

**Table 6:** Principal long-term sequelae after pediatric mandibular condylar fracture.

Long-Term Sequela	Typical Manifestations
Growth disturbance	Reduced ramus height; facial asymmetry; midline deviation; crossbite or open bite; occlusal-plane cant; mandibular hypoplasia or retrognathia
TMJ ankylosis	Fibrous or bony fusion; severe hypomobility; impaired speech, nutrition, and oral hygiene; micrognathia; possible airway compromise in bilateral disease
Persistent functional TMJ morbidity	Pain or joint symptoms; restricted maximal opening; limited lateral or protrusive excursions; mandibular deviation; impaired chewing function

**Table 7:** Suggested longitudinal assessment domains; intervals should be individualized.

Clinical Phase	Assessment Domains	Purpose
Early recovery	Pain, nutrition, comfortable opening, mandibular deviation, occlusion, appliance tolerance, and safeguarding concerns	Prevent avoidable immobility and identify unstable occlusion or treatment failure
Functional rehabilitation	Opening, excursions, joint symptoms, reproducible intercuspation, midlines, muscle function, adherence, and oral hygiene	Confirm coordinated movement and detect persistent functional shifts
Mixed dentition and growth	Eruption, arch relationships, crossbite/open bite, facial photographs, symmetry, occlusal plane, and interceptive needs	Distinguish transient adaptation from evolving asymmetry or malocclusion
Adolescence and maturity	Facial balance, stable occlusion, joint function, residual asymmetry, patient-reported concerns, and indicated imaging	Determine whether further orthodontic, reconstructive, or orthognathic evaluation is needed

A fixed universal schedule is not supported by evidence. Early rehabilitation merits closer clinical review, followed by progressively spaced growth assessments when function and occlusion remain stable. Repeat ionizing imaging should be reserved for new symptoms, clinically relevant asymmetry, suspected growth disturbance, or treatment planning.

## Data Availability

No new data were created or analyzed in this study. Data sharing is not applicable to this article.
